# Leading with purpose: Unraveling the impact of responsible leadership on employee green behavior in the workplace

**DOI:** 10.1016/j.heliyon.2024.e30096

**Published:** 2024-04-26

**Authors:** Yingdan Xiao, Xiangnan Tao, Pengyu Chen, Daisy Mui Hung Kee

**Affiliations:** a School of Business, Macau University of Science and Technology, Macau, China; b School of Business, Nanjing University, Nanjing, China; c School of Management, Universiti Sains Malaysia, Penang, Malaysia

**Keywords:** Responsible leadership, Green behavior, Stakeholder value, Felt obligation for constructive change, Superior-subordinate relationship, Positive emotion

## Abstract

Although sustainability has been a priority for organizations, there is still a lack of research on how leaders with a stakeholder perspective can motivate employees to adopt green behavior for sustainability in a complex and changing environment. This paper introduced social cognitive theory to describe two mechanisms by which responsible leadership predicts employee green behavior. Our research considers felt obligation for constructive change and stakeholder value as mediations with cognitive perspective in this process. Additionally, we consider the moderating effects of positive emotion and the superior-subordinate relationship. Our model received support from the investigation and research. By emphasizing the significance of perceived responsible leadership and proposing a new way of perceiving employee green behavior that ensures guidance from responsible leadership along the cognition perspective, the present research contributes to our understanding of the incentive effect of responsible leadership on employee green behavior.

## 1 Introduction

In recent decades, with the increase in energy costs, companies have to face up to the impact of changes in natural resources on their sustainability [[1], [2], [3]], which reminds organizations to pay attention to environmental performance while focusing on economic gains, and Improvement of environmental performance must adopt relevant management strategies [4]. However, the implementation of management decisions requires employee participation and execution, and the effectiveness of these matters often depends on employee green behavior [5]. Past researches have shown that employee green behavior can bring positive outcome, such as Del et al. [6] argue that employees engage in green behavior could enhance the competitive advantage of enterprises; furthermore, it also can positively impact on cost savings within organization [7], and from individual level, it also boost employee task performance [1]. Thus, cultivating employee green behavior is crucial to the sustainable development of the organization [8]. How to promote employee green behavior in industries such as manufacturing industries, service industries or technological industries are attracting the attention of academic and business circles [1]. Employee green behavior is defined as spontaneous and organizationally mandated actions performed by employees in the course of their work with the aim of conserving nature or enhancing the organization's environmental performance [9]. Green behavior in organizations include the following categories: sustainable work (such as using sustainable technology), avoiding harm (such as preventing pollution), saving resources (such as reusing resources), influencing others (such as spreading the concept of sustainable development) and taking positive actions (such as participating in green marches). That is, the green behavior in the workplace is more about the responsibility of environmental sustainability [10].

To better understand how to motivate employee green behavior in organization, scholars have certificated that employee green behavior can be promoted by person factors or context factors [7]. Person factors such as green behavior intention as within-person level that can effectively affect employees' pro-environmental behavior [11]; pro-environmental attitude as an between-person level promotes employee green behavior through motivational states as a mediator [12]. Contextual factors such as leadership style are an important element in predicting green behavior, as recommended by scholars [7,13]. For example, humble leadership has been shown to have a positive impact on employee green behavior, as a humble leader is likely to focus on how employees influence the leadership process [14]; Ethical leadership emphasizes leading by example and two-way communication in promoting employee green behavior [15]. Hence, the traditional leadership like these mainly focus binary relationship (leader and their subordinate), and the aim of leadership is to improve employees' positive outcomes related to green. Compared with the traditional leadership mentioned above, which emphasizes being responsible for employees in promoting green outcomes, responsible leadership goes beyond the binary relationship between leaders and followers, and promotes positive responses from employees while being responsible for the organization, society, and nature [16]. Based on the stakeholder perspective, responsible leadership is a mixture of social responsibility, ethics and leadership to positively change social and environmental goals as well as sustainable value creation goals [17]. According to its definition, responsible leadership regard natural ecosystem as a crucial stakeholder [18,19], and concentrates on social and environment-friendly sustainability to accomplish the balance among people, society and the nature [20,21]. To achieve a balance among the three, leader must first implement it into individuals within the organization. Responsible leaders have the obligation to enhance employees' understanding of stakeholders, transmit related values, and encourage them to participate in the organization's relevant green social responsibility activities. And by engaging in green behavior, employees are responding to the organization's call for green action. Therefore, one of the motivations of this study is to explore the impact of responsible leadership on employee green behavior.

Indeed, exiting studies have drawn the conclusion that responsible leadership has been found to be effective in boosting employee green behavior. For example, Yang [22] confirmed that moral reflectiveness mediate the relationship between responsible leadership and employee green behavior through the perspective of social responsibility. Younas et al. [23] believe that a shared green vision has a positive impact on how responsible leadership on in role green behavior and extra role green behavior; however, his research focused on green behavior that employees influence through the leader's description of a green vision and did not take too much account of employees' actual aspirations. Ahmed et al. [24] argue that leader identification and autonomous motivation as mechanisms can cultivate voluntary green behavior of employees; meanwhile, Zhang et al. also explored voluntary green behavior through organizational climate [3]. By summarizing the literature mentioned above, it can be found that the current research on responsible leadership on employee green behavior mainly focuses on social responsibility and the impact of the organization's internal and external environmental change reflection mechanisms, without paying much attention to the impact of employees' psychological perceptions on green behavior.

In order to fill the gap in this aspect, this article will explore how the feeling of obligation for constructive change and stakeholder values as mechanisms to investigate whether, how, and under which conditions responsible leadership may influence employee green behavior. Basing on social cognitive theory, this theoretical model interacts the effects among external environment, employee cognitive process, and employee behavior [25]. Social cognitive theory states that the external environment can directly influence an individual's subjective initiative and then shape their behavior [26]. It is believed that social cognitive theory can analyze the rationality of the theoretical model of this study, as previous research has found that responsible leadership can be considered a significant factor of the external environment that can positively influence employees' subjective cognition and behavior [27]. Specially, responsible leadership focuses on the sustainable development and positive change of nature, society, organizations and individuals, and its main purpose is to achieve balanced development among them [20,24]. Furthermore, responsible leadership not only exercises social responsibility for stakeholders but also leads by example by focusing on issues of environmentally sustainable development and actively seeking to improve green aspects [27,28]. Since the behavior of leaders who actively take responsibility for their stakeholders and strive for continuous improvement can subtly influence employee cognition and behavior through daily contact, we hypothesize that under the influence of responsible leadership, employees' felt obligation for constructive change will increase, which in turn will improve employee green behavior. At the same time, we propose that responsible leadership positively predicts stakeholder value, which may have a positive relationship with their green behavior.

**Fig. 1:**
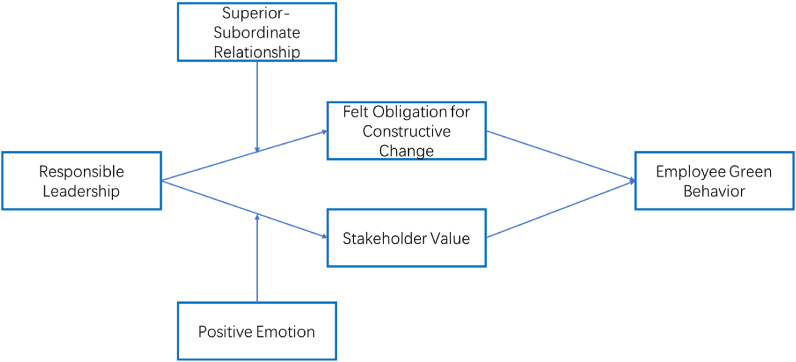
Conceptual framework.

Likewise, We further explore the potential boundary conditions of responsible leadership to individual cognitive and behavioral processes. This study introduces superior-subordinate relationship and individual positive emotion as moderators influence how responsible leadership impact on employee outcomes. Basing on social cognitive theory, the processes of environmental factors affecting individual cognition and behavior may be influenced by employees’ individual differences [25]. Hence, The relationship between supervisor and subordinates goes beyond the exchange of work relationships, including the quality of relationships in life and work [29]. It will affect the level of acceptance by employees of responsible leadership as a model from the everyday work and life aspects, including their degree of cognitive identity with and follow-up behavior. Hence, we believe that employees with high-quality superior-subordinate relationships are more likely to be subjected to the image of responsible leadership. That is, superior-subordinate relationship positively influences the relationship between responsible leadership and felt obligation for constructive change (FRCC), thus more conducive to the practice of green behavior. In addition to superior-subordinate relationship, emotion also play an important guiding role in individual cognition and behavior [30,31]. More specifically, when individuals are accompanied by high positive emotion, the extent to which they model responsible leadership is also high, including positive recognition of their values and willingness to follow responsible leadership. Therefore, we predict that employees with high positive emotion are more likely to identify with responsible leadership; that is, positive emotion positively moderates the relationship between responsible leadership and stakeholder value, which is more conducive to the practice of sustainable green behavior. The theoretical model is depicted in Fig. 1.

Our research makes several theoretical contributions to the field of green behavior research. First of all, we clarify the influence of responsible leadership on employee green behavior to broaden the literature of responsible leadership. In addition, based on social cognitive theory, this study constructs a dual mediation model, and then reveals the potential mechanism of responsible leadership affecting employee green behavior. Specifically, this paper regards the felt obligation for constructive change and stakeholder value as mediator factors that may link responsible leadership with employee green behavior. In this way, this paper can understand that responsible leadership puts forward a new perspective on employee results from a cognitive perspective. moreover, we have contributed to the current literature by exploring the boundary conditions that may limit the influence of responsible leadership on employee cognition and green behavior. Here, we study the moderating effect of the relationship between superior-subordinate relationship between responsible leadership and felt obligation of constructive change, and the moderating effect of employees' positive emotion on the direct relationship between responsible leadership and stakeholder value. In conclusion, we clarify the conditions under which responsible leadership can promote employee green behavior to the greatest extent.

## 2 Development of hypotheses

### 2.1 Responsible leadership positively impact on employee green behavior

Scholars extend the leadership style from the binary relationship between leaders and subordinates to the pluralistic relationship between leaders and stakeholders, and put forward the concept of responsible leadership [17]. Responsible leadership is defined as a leadership behavior that achieves mutual trust, cooperation and stability with different stakeholders inside and outside the organization by actively fulfilling social responsibilities, and realizes the sustainable development of business and the common interests of all parties by meeting the requirements of all stakeholders and coordinating the responsible behaviors of all parties with a shared vision [20,32]. This concept comprises three fundamental components: efficacy, which signifies that it generates a favorable impact on stakeholders; ethicality, which underscores adherence to moral and ethical principles; and sustainability, which means that the organization and its stakeholders strive for sustainable development. Based on this concept, this study will analyze how responsible leadership affects employee green behavior.

Drawing upon social cognitive theory, employees gradually internalize their leaders' values through observation and imitation their conduct during their work tasks [25]. This study posits that responsible leadership increases employee green behavior within the organization. By interacting with responsible leadership in the workplace and observing and imitating the ideas and actions of these leaders while performing tasks, subordinates can develop green behavior. Striving for harmony among individuals, organization, society, and the nature, responsible leadership is founded on stakeholder theory and incorporates the principles of ethical leadership and corporate social responsibility [17,33]. In addition to prioritizing natural ethics, it exemplifies social responsibility by setting a moral precedent that inspires personnel to be concerned with environmental matters. Responsible leadership creates a positive organizational atmosphere, encouraging staff to actively participate in environmental protection actions [5,34].

In addition, responsible leadership adheres to high standards of business ethics in environmental management, translating into workplace practices during task implementation to bolster social responsibility and conservation of the environment [18,35,36]. For instance, organizational recognition is strengthened by responsible leadership, and a greener work environment is fostered [37], aligning with subordinates' green behaviors. In summary, responsible leadership places equal emphasis on stakeholders, both internal and external, and strives to balance natural environmental interests with business concerns, influences subordinates to adopt green management practices, and encourages subordinates to adopt more green behavior. Hence, responsible leadership can be considered a condition for fostering employee green behavior. With respect to the given context, we postulate.

H1 Responsible leadership positively influence to Employee green behavior.

### 2.2 The positive pathway - felt obligation for constructive change (FRCC) as a promotion-focused cognition

On the basis of social cognitive theory, we posit that responsible leadership increases an individual's FRCC. FRCC prioritizes an individual's proactive vision and concepts for advancing objectives, refining processes, or altering established norms [34]. Responsible leadership, with its focus on stakeholders, encompassing the interplay between humanity, society, and the ecological environment [17], demonstrates a sense of responsibility and obligation manifested through modeling and actively seeking change. Furthermore, it is apparent that responsible leadership prioritizes the autonomy of its employees in their work [38]. When the work autonomy of employees is high, it enhances their organizational identification and lead to FRCC [[39], [40], [41]]. Prior research has established a positive correlation between identification with the organization and organization-valued behaviors, including work engagement, job performance, and organizational citizen behavior [42]. By engaging in these actions, responsible leadership make their subordinates more conscious of the company and foster a sense of pride in them, which can boost their sense of belonging, inspire them to work hard, and have an impact on their output [38]. Therefore, responsible leadership positively influences FRCC among employees.

The FRCC reflects workers' psychological state of accountability for their own job performance, indicating self-motivated individuals [40]. Employees who demonstrate autonomous motivation on obligation proactively endeavor to increase their own motivation and assume responsibility for the results of their work [43] and it means that FRCC may positively impact on employee green behavior. Employees are more inclined to demonstrate FRCC when they perceive their supervisory managers or leaders as responsible leaders in the workplace, given that one of the defining attributes of responsible leadership is a stakeholder perspective [44,45]. Additionally, basing on the social cognitive theory, responsible leadership participate in more green behavior, which also followed by their subordinates, that is employees' attitudes and behaviors are realized by responsible leadership [25], which means that when leaders engender a strong sense of obligation towards constructive change through activating their interest, employees are more inclined to engage in employee green behavior [34]. Therefore, the relationship between responsible leadership and green behavior is also likely to be strengthened through FRCC.

H2 Responsible leadership positively influence to Felt Obligation for Constructive Change.

H3 Felt Obligation for Constructive Change mediates the positive correction between Responsible leadership and Employee green behavior.

### 2.3 The positive pathway - stakeholder value as a promotion-focused cognition

Responsible leadership contributes to the augmentation of individuals' stakeholder value. Individuals are more likely to prioritize stakeholder groups and emphasize the criticality of social responsibility and ethics to organizational effectiveness when they follow the direction of responsible leaders [46]. Responsible leaders are envisaged as the architects of a stakeholder relationship network, embodying roles such as visionary, change agent, architect, coach, steward, servant, and more within this intricate web [17,47]. Stakeholder values refer to individuals' perception of the importance of ethics and corporate social responsibility [48]. Stakeholder value assumes that organizations should give priority to the integration and balance of interests of different stakeholders, beyond the interests of shareholders, and emphasize that ethics and social responsibility are the core of organizational effectiveness [49]. Involving stakeholders in organizational decision-making is not only an ethical imperative but also a strategic asset [50,51]. When confronted with critical decisions, employees prioritize and take into account the concerns of diverse stakeholder groups under the influence of responsible leadership. Thus, responsible leadership may exert positive influence on stakeholder value.

According to value research and integrate social cognitive theory, individual values significantly influence the attitudes and behaviors of others as subordinates can learn and imitate the values of leaders through demonstration and intuition [52]. The alignment of values with the way work is performed can enhance an employee's view of self and foster self-confidence, leading to the behavior of interpersonal altruism [46,50,51]. When employees align their work with positive self-view, they develop sense of self-confidence, fostering interpersonal altruism in the workplace and facilitating green behaviors [53]. Based on social cognitive theory, responsible leadership subconsciously influence employees through observation, learning, and summarization of experience strengthens their green behavior [54,55]. In addition, scholar suggested that the mediating role of stakeholder value becomes prominent when assessing an individual's attitude toward socially responsible and ethical behavior [56]. This implies that responsible leadership "infects" subordinates with stakeholder value. Subordinates are more likely to exhibit employee green behavior as they absorb the values of the stakeholders. Based on the above analysis, the following assumptions are put forward.

H4 Responsible leadership positively influences Stakeholder value.

H5 There is a beneficial indirect effect of Stakeholder value on Employee green behavior through Responsible leadership.

### 2.4 The moderating role of superior-subordinate relationship (SSG)

Superior-subordinate relationship refers to the informal personal relationship between the leader and subordinates outside the scope the work [57]. SSG highlighted as the most critical interpersonal dynamic in the workplace [58,59]. Particularly in organizations, employees place high value on a positive relationship with their supervisors, often resulting in increased promotions and rewards [60]. Social cognitive theory stated that the processes of environmental factors affecting individual cognition and behavior may be influenced by employees’ individual differences [25]. Basing on the fundamental principle of social cognitive theory and prior studies, this study asserts that high-quality SSG contribute to enhancing employees' FRCC.

Primarily, the establishment of excellent rapport between subordinates and superiors signifies a valuable 'pro' relationship within the organizational context of the Chinese culture's differential order pattern, emerging as a pivotal asset for employees [61]. As subordinates participate in the process of enhancing 'soft power,' they experience an augmented sense of responsibility and obligation to instigate changes in the existing status quo and it is manifested in increased accountability to supervisors' goals, thereby fostering a more profound FRCC. Secondly, a high-quality relationship between employees and supervisors serves to fortify employees' sense of security, resulting in the designation of employees as 'insiders' within the organizational framework [62]. To maintain this perception of safety, personnel willingly take on additional accountability and obligation regarding the prevailing state of affairs and the objectives of the organization. This proactive stance further strengthens FRCC. Thirdly, the cultivation of high-quality superior-subordinate relationship equips employees with increased access to valuable resources. To sum up, this means that subordinates can borrow the resources, power, influence and professional skills of leaders to promote the green power of sustainable development. In addition, research underscores that responsible leadership actively endorse and facilitate their subordinates' participation in dialogues with stakeholders, incorporating suggestions provided by employees [3,63]. This proactive leadership approach engenders employees' perception of being valued by responsible leadership, consequently augmenting their motivation to work and this heightened motivation, in turn, contributes to an enhanced awareness and recognition of their work and the overall organizational context, which may positively impact on FRCC of employees [54,55].

Therefore, in circumstances characterized by an elevated quality of the relationship between subordinates and superiors, responsible leadership will actively encourage and engage their employees. This active involvement aims to enhance employees' confidence in achieving their goals while simultaneously refining their work methodologies. The ultimate goal is to amplify the sense of obligation among subordinates, motivating them to actively participate in constructive changes. In summary, this paper puts forth the following hypotheses.

H6 The Superior-Subordinate Relationship moderates the effect of Responsible leadership on Felt Obligation for Constructive Change. That is, the relationship between Responsible leadership and Felt Obligation for Constructive Change is strengthened when the quality of the Superior-Subordinate Relationship is good.

### 2.5 The moderating role of positive emotion

Psychologists argue that emotion are an essential element in the workplace and that if managers really want to understand what influences their employees, they need to consider the emotional states of those who work for them [64]. Positive emotion is a range of positive feelings, including enthusiasm, gratitude, excitement, happiness, and pride [65].It has been demonstrated as key elements in numerous studies to impact cognitive processing, and then lead to positive individual behavior [66]. Hence, this study intents to explore how positive emotion moderates the relationship between responsible leadership and stakeholder value.

Firstly, emotional factors significantly influence the motivation of individuals to participate in particular behavior, which means that positive emotion have the function of activating general action tendencies and can promote the continuity of activities [67]. That is individual with stakeholder value exist a high level of action activation under the high level of positive emotion. In other words, under the same level of green value concept, individuals with positive environmental attitudes (people who give priority to environmental issues) are more inclined to have a positive connection with the environment and maintain the consistency of their behavior. Furthermore, individuals with positive emotion have a stronger sense of subjective control over behavior results and a higher sense of self-efficacy [68]. Therefore, when employees can't control themselves, they may stop accepting the guidance and values from their leaders and then take no further action. That is to say, when employees have high positive emotion, their acceptance may be higher when responsible leadership serve as role models to transmit stakeholder values to them. Therefore, employees can experience positive emotion in this process to enhance their stakeholder value, thus improving their green work motivation and strengthening their recognition and recognition of green work and organization.

H7 Positive emotion moderates the effect of Responsible leadership on Stakeholder value, that is the relationship between Responsible leadership and Stakeholder value can be strengthened when the level of Positive emotion is high.

The summary of hypotheses related to this study is mainly presented in Table 1.

**Table 1:** Summary of hypothesis.

H1: Responsible leadership positively influence to Employee green behavior.
H2: Responsible leadership positively influence to Felt Obligation for Constructive Change.
H3: Felt Obligation for Constructive Change mediates the positive correction between Responsible leadership and Employee green behavior.
H4: Responsible leadership positively influences Stakeholder value.
H5: There is a beneficial indirect effect of Stakeholder value on Employee green behavior through Responsible leadership.
H6: The Superior-Subordinate Relationship moderates the effect of Responsible leadership on Felt Obligation for Constructive Change. That is, the relationship between Responsible leadership and Felt Obligation for Constructive Change is strengthened when the quality of the Superior-Subordinate Relationship is good.
H7: Positive emotion moderates the effect of Responsible leadership on Stakeholder value, that is the relationship between Responsible leadership and Stakeholder value can be strengthened when the level of Positive emotion is high.

## 3 Methods

### 3.1 Procedures, samples, and data collection

As a pivotal driver of China's economic growth, the Guangdong-Hong Kong-Macao Greater Bay Area's strategies and practices in green development hold immense significance for China's green transformation and global sustainability efforts. Similarly, the northwest and southwest regions of China hold special significance and pivotal roles in the nation's development agenda. Investigating the green behavior of employees in these regions can offer valuable insights for shaping local green development policies. Moreover, through cross-regional comparisons, we can identify both commonalities and differences in the impact of employees' green behavior across diverse regions. This study primarily focuses on private enterprises in selected regions, encompassing various industries such as manufacturing, services, agriculture, and technology. The diversity of industries enables a more comprehensive understanding of employees' green behavior across different regional and industrial contexts, including varying green management practices and employee behavior patterns. Hence, a sample of full-time employees employed in various sectors for two months within the Greater Bay Area, Guangdong-Hong Kong-Macao region, the northwest and southwest regions of China was utilized for this study. Industries surveyed include manufacturing, agriculture, services, real estate and technology industries. To ensure the generalizability of the findings and avoid contextual limitations specific to a single organization, participants were deliberately selected from various industries. A web-based survey, comprising a series of well-orchestrated steps, was employed to collect data. Initially, the managers of each organization were contacted to assure them that the survey would be used for academic purposes only, ensuring complete anonymity and non-disclosure of any confidential company information.

As a goodwill gesture, researchers offered to share valuable insights derived from the survey analysis with the participating companies. Subsequently, close collaboration was established with company managers to designate a coordinator within each organization. These coordinators were primarily responsible for distributing the survey questionnaire among the company's employees. Comprehensive briefings about the questionnaire's purpose and the strict maintenance of respondent confidentiality were provided to company managers, ensuring that no personal information would be disclosed. Finally, the online questionnaire was distributed to designated coordinators, who administered it to their respective subordinates. Upon completion of the survey by all participating employees, researchers diligently collected and systematically organized the data for subsequent analysis, leading to the creation of the final dataset.

In order to reduce the potential impact of common method bias [69]. Data were collected twice from employees. The first data collection point (T1) involved employees submitting a computerized questionnaire. Within this questionnaire, employees assessed control variables, responsible leadership, stakeholder value, Felt Obligation for Constructive Change, SSG, positive emotion and provided detailed information about their demographic characteristics. In this stage, we totally received 1211 responses. After completing the survey, personnel are granted permission to disconnect from the account through which they had previously established a connection. The survey team initiated the processing of the employee's initial questionnaire once it had been received. One month subsequent to the initial data collection point (T2), the second data collection point (T2) was once more executed through the utilization of an electronic questionnaire (T1). Participants assessed their own employee green behavior in this survey. In this stage, we finally received 1055 responses. After removing the responses that took less than half the average time to complete the questionnaire, randomly selected an option and chose the wrong choice for the attention check item, we got 1002 valid data with a response rate of 82.7 %. This sample size surpasses the minimum threshold of 50 responses that is recommended for each latent variable, is considered more than sufficient for thorough data analysis. Further, the ethical approval for underlying research has obtained from Macau University of Science and Technology Research Ethics Review Committee.

### 3.2 Measurement

All variables, unless otherwise specified, were evaluated utilizing a five-point Likert scale where five indicates strong agreement and one indicates strong disagreement. Furthermore, all materials were provided in the Chinese language, and any English components were back-translated into Chinese in accordance with standard practices. Hair Jr et al. [70] suggested that scores exceeding 0.60 are acceptable in various contexts. Voegtlin [33] devised a five-item scale for assessing responsible leadership, ranging from 1 (indicating strong agreement) to 5 (strongly disagree). Zhang et al. [71] developed a thirteen-item scale for measuring employee green behavior, which ranged from 1 (strongly agree) to 5 (strongly disagree). Shafer et al. [72] devised an eight-item scale for measuring stakeholder value, ranging from 1 (strongly agree) to 5 (strongly disagree). The Felt Obligation for Constructive Change is determined using a 5-item scale developed by Liang et al. [73], with items ranging from 1 (strongly agree) to 5 (strongly disagree). Law et al. [57] devised a six-item scale for measuring SSG, which ranged from 1 (strongly agree) to 5 (strongly disagree). Five items derived from Menge [74]were used to assess positive emotion on a five-point Likert scale ranging from 1 (strongly agree) to 5. (strongly disagree). This study only adapts the items, tested through academically accepted methods based on the preceding literature. According to Table 2, the Cronbach's alpha coefficients for the following variables: responsible leadership, employee green behavior, stakeholder value, Felt Obligation for Constructive Change, superior subordinate relationship, and positive emotion, which are all 0.87, 0.91, 0.89, 0.92, 0.85, and 0.87, are all acceptable.

**Table 2:** Sources of measures items and Cronbach's alpha of the factors.

Constructs	No. Of items	Cronbach's Alpha
**RL**	5	0.87
**EGB**	13	0.91
**FRCC**	5	0.92
**PE**	5	0.87
**SSG**	6	0.85
**SV**	8	0.89

Notes: RL = responsible leadership; EGB = employee green behavior; SV = stakeholder value; FRCC=Felt Obligation for Constructive Change; SSG = superior subordinate relationship; PE = positive emotion.

### 3.3 Control variable

Certain demographic factors, according to Abrahamse and Steg [75], may influence individual green behaviors. Demographic variables include gender, age, education, industry [76,77].We controlled for gender (1 for male,0 for female) due to the potential gender bias in rating of employee green behavior [5]. Industries (Technology, Service, Retail and Agriculture) were controlled for as it may affect people from different industries have different views on green behavior. Educational level (below junior college, junior college, bachelor degree, and postgraduate studies) was controlled for as it may influence the domain-relevant knowledge that is essential for green behavior [8,78]. In order to clearly reflect the reflection of control variables in this study, the categorization of industry, education level, and age is a focal point of this study, which has been recorded using dummy variables. When defining dummy variables, we base our definitions on the number of categories within the categorical variables. For example, if there are k categories, then k-1 dummy variables are created to avoid issues of multicollinearity in linear regression analysis. In addition, according to statistics, 59.4 % were male, while 40.6 % were female. Educational levels were diverse, with 5.33 % below junior college (secondary education or less), 17.5 % with Junior College (Associate degree), 66.5 % holding a Bachelor's degree, and 110.5 % having pursued Postgraduate studies (Master's degree and above). Industry distribution showcased diversity, encompassing 20.8 % from the service industry and 21.8 % from the technology and information fields, manufacturing sector (11.7 %) and retail (4.8 %). Furthermore, industry including another field is 38.8 %.

### 3.4 Model testing

The model test consists of two main steps [79]. The measurement model was initially validated through confirmatory factor analysis (CFA). Additionally, the discriminant validity of the latent variables was assessed by the researchers. To examine common method bias, one-factor test and common latent factor test were applied (CMB). In order to assess the resilience of the benchmark model, a number of additional models were juxtaposed with it [80]. Additionally, in order to examine the hypotheses, SPSS and structural equation modeling (SEM) were implemented. The chi-square, degrees of freedom (df), and corresponding p-values of the model are provided. In order to assess the adequacy of the model, several fit indices were implemented: the comparative fit index (CFI >0.90), the Tucker-Lewis Index (TLI >0.90), the Incremental Fit Index (IFI >0.90), and the Root Mean Square of Error Approximation (RMSEA <0.08). The CFA and SEM methodologies were assessed utilizing AMOS v.24. Using one thousand bootstrap samples, confidence intervals (CIs) were calculated to examine indirect effects. Furthermore, the correlations, means, and standard deviations among the variables are calculated utilizing SPSS 23.0. The outcomes of these calculations are presented in Table 5.

**Table 3:** Average variance extracted, maximum shared variance, maximal reliability, and composite reliability among study factors.

	CR	AVE	MSV	MaxR(H)	RL	EGB	SV	FRCC	PE	SSG
**RL**	0.918	0.693	0.310	0.923	**0.832**					
**EGB**	0.952	0.606	0.194	0.953	0.440	**0.779**				
**FRCC**	0.943	0.767	0.557	0.946	0.553	0.243	**0.779**			
**PE**	0.945	0.774	0.538	0.949	0.525	0.243	0.733	**0.876**		
**SSG**	0.941	0.727	0.392	0.946	0.557	0.243	0.626	0.6613	**0.880**	
**SV**	0.923	0.600	0.557	0.931	0.531	0.239	0.747	0.624	0.524	**0.775**

Notes: MaxR (H) = maximal reliability; RL = responsible leadership; EGB = employee green behavior; SV = stakeholder value; FRCC=Felt Obligation for Constructive Change; SSG = superior subordinate relationship; PE = positive emotion.

**Table 4:** Confirmatory factor analysis.

Model	Constructs Combined	x^2^	df	x^2^/df	RMSEA	TLI	NFI	CFI
Model1	RL, SV, FRCC, PE, SSG, EGB	2684.417	802	3.347	0.048	0.925	0.903	0.930
Model2	RL, SV, FRCC, PE + SSG, EGB	4288.574	807	5.314	0.066	0.862	0.845	0.870
Model3	RL, SV, FRCC + PE + SSG, EGB	6089.563	811	7.509	0.081	0.791	0.780	0.803
Model4	RL, SV,+FRCC + PE + SSG, EGB	7749.399	814	9.520	0.092	0.727	0.720	0.742
Model5	RL, SV + FRCC + PE + SSG,+EGB	12512.075	817	15.315	0.120	0.541	0.548	0.564
Model6	RL + SV + FRCC + PE + SSG + EGB	14183.187	818	17.339	0.128	0.476	0.488	0.502

Notes: **x**^**2**^**/df** represents the differences between the other models and the 6-actor model; the acronyms CFI, NFI, and TF1 stand for comparative fit index, Normaled fit index, and Tucker-Lewis index, respectively. Model 1 served as the baseline model for the computation of Models 2, 3, 4, 5, and 6. RMSEA = root mean square error of approximation; RL = responsible leadership; EGB = employee green behavior; SV = stakeholder value; FRCC=Felt Obligation for Constructive Change; SSG = superior subordinate relationship; PE = positive emotion.

**Table 5:** Means, SDs. and correlations.

	M	SD	1	2	3	4	5	6	7	8	9	10
Gender	1.41	0.49	1									
Age	1.47	0.80	0.231**	1								
Degree	2.83	0.68	0.099**	−0.180**	1							
Industry	4.49	1.55	−0.101**	−0.026	−0.049	1						
RL	4.20	0.61	−0.008	0.058	−0.077*	−0.062*	1					
SV	4.43	0.52	−0.032	0.056	−0.049	−0.027	0.393**	1				
FRCC	4.32	0.61	−0.025	0.043	−0.094**	−0.025	0.410**	0.611**	1			
PE	4.43	0.51	−0.023	0.003	−0.047	−0.006	0.366**	0.573**	0.552**	1		
SSG	4.00	0.66	−0.018	0.025	−0.122**	−0.036	0.372**	0.502**	0.558**	0.519**	1	
EGB	4.23	0.49	−0.019	0.051	−0.076*	0.035	0.273**	0.325**	0.286**	0.303**	0.304**	1

Notes: RL = responsible leadership; EGB = employee green behavior; SV = stakeholder value; FRCC=Felt Obligation for Constructive Change; SSG = superior subordinate relationship; PE = positive emotion; N = 1102.*p < 00.05; **p < 00.01.

## 4 Results

The results of the CFA indicated that the six-factor model proposed, consisting of responsible leadership, employee green behavior, stakeholder value, Felt Obligation for Constructive Change, superior subordinate relationship, and positive emotion, adequately described the data (Δχ^2^ = 3.3，p < 0.001, CFI = 0.93, TLI = 0.925, RMSEA = 0.048). The maintenance of content validity was ensured through the construction of questionnaire instruments that were founded on theoretical concepts derived from academic literature. Additionally, the questions' representativeness and suitability were confirmed by a panel of academic and professional experts, which comprised two management academics, one built environment expert, and one academic HR expert. Incorporating panel of experts and pilot study feedback, the ultimate iteration of the questionnaire was formulated. In order to ensure criterion validity, published assessments were utilized to obtain employee responses. In this study, published measures were utilized. By utilizing factor loadings, composite reliability (CR), average variance extracted (AVE), and maximum shared squared variance, the convergent validity of the measurement model was examined (MSV). Additionally, the model's discriminant validity was evaluated [81].

The AVE values exceed 0.5 for responsible leadership, employee green behavior, stakeholder value, Felt Obligation for Constructive Change, superior subordinate relationship, and positive emotion, that can be accepted [70]. The discriminant validity was calculated using MSV as follows: (square root of AVE > inter-construct correlations; MSV < AVE). Discriminant validity exceeded 0.7 for all constructs (as shown in Table 3) [70].

In summary, the results corroborated the construct validity and reliability of the measurement model, as presented in Table 4. According to introduction by Podsakoff et al. [82], CMB did not pose a concern in the present study. The SEM model was derived subsequent to the CFA. Assess the resilience of the foundational model. In Table 3, 6-factor, 5-factor, 4-factor, 3-factor, 2-factor, and 1-factor models were compared to the baseline model for the purposes of this study at the CFA stage. The baseline model consistently exhibited superior values for chi-square, df, x^2^/df, CFI, NFI, TLI, and RMSEA when compared to the competing models. Thus, Model 1 was determined to be the most reliable in terms of hypothesis testing.

### 4.1 Hypothesis test

The result of the hierarchical regression of how RL on EGB is presented in Table 6. According to model 2, the R-squared is 0.187, indicating that the explainable variation of employee green behavior is 18.7 % and that RL has a significant positive effect on EGB (β = 0.366, p < 0.001), supporting hypothesis 1. According to Model 2 of Table 7, the R-squared is 0.301, indicating that the explainable variation of FRCC is 30.1 % and that RL has a significant positive effect on FRCC (β = 0.429, p < 0.001), supporting hypothesis 2. According to Model 2 of Table 8, the R-squared is 0.284, indicating that the explainable variation of SV is 28.4 % and that RL has a significant positive effect on SV (β = 0.385, p < 0.001), supporting hypothesis 4.

**Table 6:** Responsible leadership to Employee green behavior.

			EGB					
			M1			M2		
			**β**	t	P-Value	**β**	**t**	**P-Value**
**Control Variables**		Gender (male)	0.007	0.201	0.841	0.005	0.158	0.875
	Industry	manufacture	0.077	1.496	0.135	0.033	0.660	0.509
		agriculture	−0.058	−0.542	0.588	−0.055	−0.532	0.595
		service	−0.187	−4.450	0.000	−0.180	−4.440	0.000
		retail	−0.073	−0.983	0.326	−0.073	−1.021	0.307
		Technology	−0.140	−3.379	0.000	−0.126	−3.155	0.002
	Age	Age29-35	0.019	0.489	0.625	−0.004	−0.107	0.915
		Age36-40	0.102	1.263	0.207	0.076	0.968	0.333
		Age41-50	0.101	1.375	0.169	0.086	1.203	0.229
	Educational level	junior	−0.033	−0.427	0.670	−0.038	−0.504	0.614
		bachelor	−0.032	−0.448	0.654	−0.034	−0.484	0.628
		postgraduate	−0.154	−1.840	0.066	−0.116	−1.425	0.155
**IDV**		RL				0.366	13.398	0.000
		R^2^	0.044			0.106		
		Adjust R^2^	0.032			0.094		
		F	3.790		0.000	9.027		0.000

Notes: **IDV** stand for independent variable. RL = responsible leadership; EGB = employee green behavior; SV = stakeholder value; FRCC=Felt Obligation for Constructive Change; SSG = superior subordinate relationship; PE = positive emotion, *p < 0.05; **p < 0.01; ***p < 0.001.

**Table 7:** Responsible leadership to Felt Obligation for Constructive Change.

			FRCC					
			M1			M2		
			**β**	t	P-Value	**β**	**t**	**P-Value**
**Control Variables**		Gender (male)	0.010	0.246	0.806	0.007	0.185	0.853
	Industry	manufacture	0.175	2.782	0.006	0.093	1.588	0.113
		agriculture	0.134	1.018	0.309	0.139	1.153	0.249
		service	−0.172	−3.342	0.000	−0.160	−3.372	0.000
		retail	−0.105	−1.158	0.247	−0.106	−1.267	0.205
		Technology	−0.176	−3.463	0.000	−0.151	−3.217	0.001
	Age	Age29-35	0.025	0.527	0.598	−0.018	−0.417	0.677
		Age36-40	0.036	0.360	0.719	−0.014	−0.154	0.878
		Age41-50	0.148	1.639	0.102	0.119	1.427	0.154
	Educational level	junior	−0.018	−0.193	0.847	−0.027	−0.311	0.756
		bachelor	−0.019	−0.218	0.828	−0.022	−0.270	0.787
		postgraduate	−0.240	−2.325	0.020	−0.167	−1.757	0.079
**IDV**		RL				0.388	13.409	0.000
		R^2^	0.056			0.201		
		Adjust R^2^	0.044			0.191		
		F	4.872		0.000	19.141		0.000

Notes: **IDV** stand for independent variable. RL = responsible leadership; EGB = employee green behavior; SV = stakeholder value; FRCC=Felt Obligation for Constructive Change; SSG = superior subordinate relationship; PE = positive emotion, *p < 0.05; **p < 0.01; ***p < 0.001.

**Table 8:** Responsible leadership to Stakeholder value.

			SSV					
			M1			M2		
			**β**	t	P-Value	**β**	**t**	**P-Value**
**Control Variables**		Gender (male)	0.035	0.999	0.318	0.033	1.000	0.317
	Industry	manufacture	0.173	3.167	0.002	0.104	2.049	0.041
		agriculture	0.022	0.197	0.844	0.027	0.257	0.797
		service	−0.124	−2.782	0.006	−0.114	−2.754	0.006
		retail	−0.145	−1.840	0.066	−0.146	−1.993	0.046
		Technology	−0.115	−2.614	0.009	−0.094	−2.301	0.022
	Age	Age29-35	0.073	1.809	0.071	0.038	1.007	0.314
		Age36-40	0.001	0.013	0.990	−0.040	−0.503	0.615
		Age41-50	0.160	2.037	0.042	0.135	1.861	0.063
	Educational level	junior	0.079	0.966	0.334	0.072	0.946	0.344
		bachelor	0.067	0.876	0.381	0.065	0.913	0.361
		postgraduate	−0.030	−0.334	0.739	0.031	0.368	0.713
**IDV**		RL				0.322	12.737	0.000
		R^2^	0.046			0.180		
		Adjust R^2^	0.034			0.170		
		F	3.954		0.000	16.724		0.000

Notes: **IDV** stand for independent variable. RL = responsible leadership; EGB = employee green behavior; SV = stakeholder value; FRCC=Felt Obligation for Constructive Change; SSG = superior subordinate relationship; PE = positive emotion, *p < 0.05; **p < 0.01; ***p < 0.001.

The mediation test uses Smart-pls 4.0. According to Hair et al. [81] 's bootstrap approach, and the report of this results refers to the test result reporting method of Cole et al. [83]. Table 9 presents an impact analysis of FRCC and SV between RL and EGB, the indirect effect of FRCC Bootstrap 95 % confidence interval is that LLCI (lowest value) and ULCI (highest value) do not contain 0 (Bootstrap CI = [0.001,0.069]), which helps to have a mediating effect, responsible leadership can not only directly predict employee green behavior, but also through the mediational effect of FRCC. Consequently, hypothesis 3 is validated. Table 9 also demonstrates the impact analysis of SV between RL and EGB, the indirect effect of SV Bootstrap 95 % confidence interval is that LLCI (lowest value) and ULCI (highest value) do not contain 0 (Bootstrap CI = [0.045, 0.112]), which helps to have a mediating effect, responsible leadership can not only directly predict employee green behavior, but also through the mediational effect of SV. Therefore, hypothesis 5 is supported.

**Table 9:** The role of FRCC and SV as mediation.

	Effect	SE	t	p	LLCI	ULCI
RL-FRCC-EGB (Indirect effect)	0.034	0.017	2.009	0.000	0.001	0.069
RL-SV-EGB (Indirect effect)	0.077	0.017	4.552	0.045	0.045	0.112

Notes: N = 1002. RL = responsible leadership; EGB = employee green behavior; SV = stakeholder value; FRCC=Felt Obligation for Constructive Change; SSG = superior subordinate relationship; PE = positive emotion, *p < 0.05; **p < 0.01; ***p < 0.001.SE, standard errors; CI, confidence interval; P, unstandardized regression coefficients. 1000 resamples served as the basis for the analysis. The 95 percent confidence intervals are enclosed in square brackets. ***p < 0.001, **p < 0.01, and *p < 0.05.

**Fig. 2:**
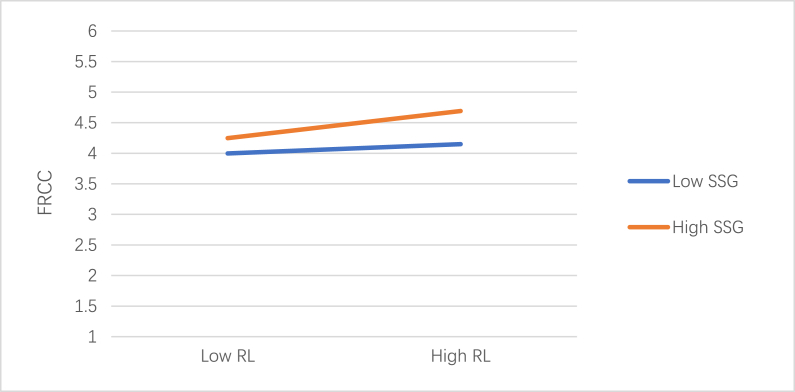
Moderating effect of SSG on the relationship of RL and FRCC.

As shown in Table 10, the examination of the manner in which SSG influences the correlation between RL and FRCC is moderating. Model 4 indicates that RL x SSG (β = 0.077, p < 0.01) had a statistically significant positive effect, suggesting that SSG modifies RL in FRCC in a significant and positive way. The results of the sample slope test, as illustrated in Fig. 2, suggested that the correlation between FRCC and RL was more pronounced during periods of high SSG compared to low SSG. These findings thus lend support to hypothesis 6.

**Table 10:** The role of SSG as moderator.

	FRCC							
	M1		M2		M3		M4	
	β	t	β	t	β	t	β	t
Gen(M)	0.01	0.27	0.01	0.29	0.01	0.19	0.02	0.49
Age	0.04	1.53	0.02	1.02	0.02	1.07	0.02	1.05
Manu	0.18	2.79	0.10	1.62	0.06	1.02	0.05	0.87
Agr	0.13	0.97	0.13	1.06	0.10	0.87	0.10	0.90
Service	−0.17***	−3.35	−0.16***	−3.39	−0.12**	−2.73	−0.12*	−2.61
Retail	−0.11	−1.18	−0.11	−1.30	−0.04	−0.50	−0.03	−0.41
Tec	−0.18*	−3.54	−0.16*	−3.37	−0.09	−1.96	−0.08	−1.90
B bac	0.25*	2.44	0.18	1.93	0.08	0.91	0.08	0.87
Bachelor	0.22***	3.00	0.15	2.13	0.09	1.46	0.09	1.40
Post	0.22***	3.50	0.15	2.55	0.10	1.81	0.10	1.84
RL			0.23***	13.14	0.154***	8.89	0.148***	8.58
SSG					0.22***	12.24	0.20***	10.41
RL X SSG							0.07***	3.47
R2	0.06		0.20		0.30		0.31	
Adjust R2	0.05		0.19		0.29		0.30	
F	5.81***		21.89***		35.56***		34.10***	

Notice: RL = responsible leadership; EGB = employee green behavior; SV = stakeholder value; FRCC=Felt Obligation for Constructive Change; SSG = superior subordinate relationship; PE = positive emotion; Age was measured in years. Gender was coded 1 for males and 0 for females. Manu = manufacture; Agr = agriculture; Tec = technology; B bac = below bachelor; Post = postgraduate; *p < 0.05; **p < 0.01; ***p < 0.001.

**Fig. 3:**
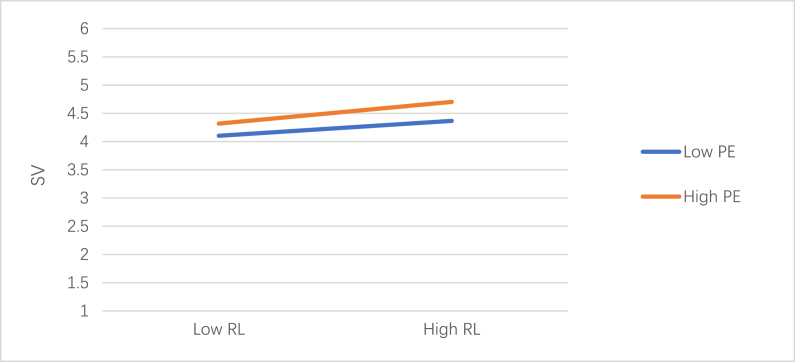
PE's moderating influence on the RL and SV.

According to Table 11, Model 4 indicates that RL x PE (β = 0.03, p < 0.05) had a statistically significant positive effect, suggesting that PE modifies RL in SV in a significant and positive way. The results of the sample slope test, as illustrated in Fig. 3, suggested that the correlation between SV and RL was more pronounced during periods of high PE compared to low PE. These findings thus lend support to hypothesis 7.

**Table 11:** The role of PE as moderator.

	SV							
	M1		M2		M3		M4	
	β	t	β	t	β	t	β	t
Gen(M)	0.03	0.96	0.03	1.03	0.03	0.90	0.03	0.98
Age	0.047*	2.13	0.035*	1.70	0.02	1.18	0.02	1.06
Manu	0.175**	3.20	0.110*	2.15	0.102*	2.08	0.101*	2.05
Agr	0.01	0.12	0.01	0.14	0.02	0.23	0.03	0.29
Service	−0.13**	−2.81	−0.11**	−2.79	−0.102*	−2.56	−0.103*	−2.59
Retail	−0.15	−1.85	−0.148*	−2.00	−0.11	−1.51	−0.10	−1.41
Tec	−0.12**	−2.65	−0.10	−2.41	−0.07	−1.68	−0.06	−1.61
B bac	0.04	0.45	−0.01	−0.17	−0.02	−0.30	−0.03	−0.39
Bachelor	0.12	1.83	0.06	0.94	0.05	0.85	0.05	0.82
Post	0.10	1.86	0.05	0.86	0.04	0.76	0.04	0.72
RL			0.18***	11.98	0.17***	11.38	0.16***	10.86
PE					0.14***	9.28	0.14***	9.29
RL X PE							0.03*	1.98
R2	0.06		0.20		0.30		0.31	
Adjust R2	0.05		0.19		0.29		0.30	
F	5.81***		21.89***		35.56***		34.10***	

Notice: RL = responsible leadership; EGB = employee green behavior; SV = stakeholder value; FRCC=Felt Obligation for Constructive Change; SSG = superior subordinate relationship; PE = positive emotion; Age was measured in years. Gender was coded 1 for males and 0 for females. Manu = manufacture; Agr = agriculture; Tec = technology; B bac = below bachelor; Post = postgraduate; *p < 0.05; **p < 0.01; ***p < 0.001.

## 5 Discussion

Based on the background of China, this paper discusses the antecedent research of employee green behavior, with a particular focus on the Guangdong-Hong Kong-Macao Greater Bay Area, southwest, and northwest regions. This focus is driven by the national policy and sustainable development strategy of Chinese. According to social cognitive theory, we developed and tested a conceptual model accounting for how responsible leadership evokes employee green behavior. We assume that responsible leadership arouses employee green behavior through dual-mediation. The outcomes demonstrated that responsible leadership was positively related to felt obligation for constructive change and stakeholder value. Both felt obligation for constructive change and stakeholder value significantly promoted their employee green behavior. Besides, we also found that mediation effects of felt obligation for constructive change and stakeholder value in the relationship between responsible leadership and employee green behavior. There is no deny that this study found that "necessity" and "sense of identity" are important mechanisms for responsible leadership to influence employee green behavior, and expounded the significance of cognitive understanding to solve the influence of leadership style on individual behavior.

Furthermore, the results show that superior-subordinate relationship from Chinese context that magnified the positive effect of responsible leadership and felt obligation for constructive change. The superior-subordinate relationship in China mainly includes close contact with leaders outside their working relationship [29], that is to say, when superior-subordinate relationship is high, responsible leadership can consider employees from many aspects and thus be more felt obligation for constructive change for employees. Besides, the results also show that positive emotion moderates the relationship between responsible leadership and stakeholder value. positive emotion encompass feelings of excitement, pleasure, and enthusiasm [66]. Responsible leadership motivates their subordinates to engage in discussions with stakeholders and adopt suggestions from employees, thereby enabling employees to augment their stakeholder values through high positive emotional experiences. This, in turn, leads to heightened work motivation and an enhanced recognition of both the work and the organization.

### 5.1 Theoretical contributions

Three significant theoretical contributions are made by this study. Firstly, it enhances the realms of social cognitive theory by providing evidence that responsible leadership can exert reinforcing effects on employee green behavior. Additionally, it responds to the inquiries of prior scholars investigating how responsible leadership influences employee green behavior [1,3].

Secondly, this study revealed the underlying mechanism that could explain the influence of responsible leadership on employee green behavior by testing the mediating effects of stakeholder value and felt obligation for constructive change. Existing literature has demonstrated that the current research on responsible leadership on employee green behavior mainly focuses on social responsibility and the impact of the organization's internal and external environmental change reflection mechanisms, without paying much attention to the impact of employees' psychological perceptions on green behavior. To bridge this gap, our study identifies stakeholder value and felt obligation for constructive change as potential mediators linking responsible leadership to employee green behavior. Our findings indicate that employees tend to internalize the values espoused by responsible leadership, leading to heightened environmental responsibility and a greater propensity for green behavior [24]. Furthermore, under the influence of responsible leadership, employees extend their considerations beyond the organization, embracing a broader array of stakeholders, which, in turn, fosters green behavior in their daily lives. This study offers valuable insights into why certain employees exhibit greener behaviors and underscores how responsible leaders can facilitate this by altering employees' perceptions of sustainable practices.

Thirdly, this study delves into the contextual conditions shaping both pathways influencing subordinate green behavior. In the examination of the stakeholder pathway, positive emotion is introduced as a potential factor impacting green behavior. positive emotion has the potential to shape subordinates' intentions towards green behavior, fostering a more positive inclination. In the exploration of the constructive change pathway through the sense of obligation, the variable of superior-subordinate-relationship is introduced. With regard to the humanistic society of China, the superior-subordinate relationship remains a pivotal interpersonal connection within any organizational culture. This introduces necessity aspect to the relationship, aligning with the cultural context. These two paths contribute two contextual conditions—one introducing "positive emotion" to evoke a more positive inclination, and the other introducing "subordinate relationships" to evoke a more obligatory inclination. Considering the intertwined nature of emotion and cognition, as highlighted by Ref. [58], this study recognizes their mutual influence on subordinates' perceptions and, consequently, their green behaviors. This dual-path, dual-context approach to responsible leadership offers a comprehensive framework for influencing subordinate green behaviors, contributing to the richness of related research frameworks and content.

### 5.2 Practical implications

This study provides organizations and individuals with four practical implications.

First and foremost, this study introduces an exploratory approach to fostering employees' green behavior. By examining two avenues—stakeholder values (emphasizing active and spontaneous identification and motivation) and a constructive sense of responsibility for change (involving passive moral obligation and a sense of responsibility)—the research demonstrates that both pathways effectively promote employees' green behavior and contribute to a broader understanding of sustainable development. This finding underscores the significance of enhancing employees' awareness of environmental issues, aligning with prior research indicating that some employees may harbor anti-green behavior attitudes. Hence, to advance sustainable development and uphold the equilibrium among individuals, organizations, society, and nature, responsible leaders must assume an exemplary role. Furthermore, leaders can share business cases pertinent to green behavior, illustrating the importance of environmental issues and endeavoring to foster a positive attitude toward green behavior among employees.

Second, give full play to the catalytic role of leadership and strengthen the training and selection of responsible leadership. Through the study of the mechanisms of influence of responsible leadership on employee green behavior, enterprises in human resources management practice should strengthen the attention to responsible leadership, and through talent cultivation to promote the formation and reproduction of responsive leadership. Enterprises should also develop appropriate standards in the recruitment selection, post promotion mechanism, screening the selection of employees with responsibility leadership characteristics, especially stakeholder values into the management class. Develop training and leadership related courses, emphasize the shaping and construction of stakeholders related values, awaken the need for green behavior, through training to cultivate responsible leadership.

Thirdly, this study serves as a reference manual for enterprises aiming to establish and nurture green environmental protection teams. The research validates the moderating influence of employees' positive emotion. Thus, it is imperative to provide employees with more encouragement in the workplace, alongside organizing activities centered around green or sustainable development themes. Additionally, the study highlights the moderating role of the superior-subordinate relationship. This finding corroborates prior research, suggesting that a positive relationship between superiors and subordinates enhances the impact of leaders on subordinates' behavior [84]. Consequently, to foster employee green behavior, organizations can facilitate opportunities for leaders and employees to enhance communication and collaboration. For instance, hosting relevant experience-sharing meetings. Leaders should endeavor to cultivate a long-term cooperative relationship with employees and offer professional assistance or information whenever needed, thereby bolstering trust and support and fostering the formation of effective green environmental protection teams.

### 5.3 Limitations and suggestions for future research

While this study possesses several strengths, such as its exploration of dual paths to elucidate the impact of responsible leadership on employee green behavior, it is not without limitations. Firstly, the longitudinal research focused on employees lacks comprehensive cross-level investigation. Future research stands to gain by delving into inter-level relationships, incorporating variables at team and organizational levels. For instance, exploring the regulatory effects of organizational culture and the green climate on responsible leadership and green behavior, and examining their relationships post-controlling for external factors. Such endeavors would augment both theoretical understanding and practical applications in this domain [3,7].

Secondly, our research was conducted in China, a nation where individuals exhibit a relatively strong natural orientation [5]. Future investigations should take into account the potential impact of cultural variations to broaden the applicability of our findings. For instance, exploring the effects of green behavior in Western and African countries could provide valuable insights. Thirdly, our study primarily examines the influence of responsible leadership on employees' green behavior from a cognitive perspective. In subsequent research, delving into the relationship mechanism from various other dimensions could prove fruitful. For example, investigating strategic perspectives such as corporate environmental strategy could offer additional insights into the dynamics at play.

## Data availability statement

Data will be made available on request.

## Ethics declarations

Review and/or approval by an ethics committee was not needed for this study because it does not involve human experimentation.

## CRediT authorship contribution statement

**Yingdan Xiao:** Writing – original draft, Funding acquisition, Data curation, Conceptualization. **Xiangnan Tao:** Visualization, Supervision, Project administration, Methodology. **Pengyu Chen:** Writing – review & editing, Writing – original draft, Validation, Methodology, Investigation, Conceptualization. **Daisy Mui Hung Kee:** Supervision, Conceptualization.

## Declaration of competing interest

The authors declare that they have no known competing financial interests or personal relationships that could have appeared to influence the work reported in this paper.
